# Chick Early Amniotic Fluid Alleviates Dextran-Sulfate-Sodium-Induced Colitis in Mice via T-Cell Receptor Pathway

**DOI:** 10.3390/antiox14010051

**Published:** 2025-01-04

**Authors:** Fan Chen, Yining Zhao, Yanfa Dai, Ning Sun, Xuezheng Gao, Jiajun Yin, Zhenhe Zhou, Ke-jia Wu

**Affiliations:** 1 Department of Psychiatry, The Affiliated Wuxi Mental Health Center of Jiangnan University, Wuxi 214151, China; 7232808001@stu.jiangnan.edu.cn (F.C.); gaoxzh007@sina.com (X.G.); yinjiajun@jiangnan.edu.cn (J.Y.); 2 Wuxi School of Medicine, Jiangnan University, Wuxi 214082, China; 6232803013@stu.jiangnan.edu.cn (Y.Z.); 6222803020@stu.jiangnan.edu.cn (Y.D.); sunning@jiangnan.edu.cn (N.S.)

**Keywords:** ulcerative colitis, inflammatory bowel disease, chick early amniotic fluid, TCR signaling pathway, tyrosine protein kinase

## Abstract

Ulcerative colitis (UC) is a chronic immune disease that is difficult to cure. We recently found that chick early amniotic fluid (ceAF) has notable anti-inflammatory and antioxidative properties, through its active components. This study demonstrates the potential of ceAF as a protective agent against UC. UPLC-MS mass spectrometry identified key components of ceAF, including various fatty acids and nucleosides. In vitro, ceAF improved viability in DSS-induced Caco-2 cells, reduced pro-inflammatory cytokines IL-1β and TNF-α, and increased the anti-inflammatory cytokine IL-10. It also upregulated the tight junction proteins ZO-1 and occludin. In DSS-induced UC mice, ceAF treatment alleviated weight loss, colon shortening, and disease activity, while improving histopathology, crypt depth, and colonic fibrosis. Mechanistically, ceAF’s anti-inflammatory effects are mediated by inhibiting the overactivation of TCR signaling through the LCK/ZAP70/LAT pathway. Our findings suggest that ceAF could be a valuable nutritional intervention for UC, potentially enhancing existing functional foods aimed at managing this condition.

## 1. Introduction

Ulcerative colitis (UC) is a chronic autoimmune disorder predominantly affecting individuals aged 30 to 40. Clinically, UC is characterized by abdominal pain, diarrhea, purulent and bloody stools, and a sensation of incomplete evacuation. The disease often follows a prolonged course, necessitating lifelong management [1]. Current therapeutic strategies, including aminosalicylates, corticosteroids, immunosuppressants, antibiotics, and biologics, primarily alleviate symptoms but fail to restore intestinal barrier integrity. These treatments are also associated with considerable side effects, underscoring the need for non-toxic functional foods (FFs) as adjunctive therapies [2].

Natural compounds and traditional Chinese medicine have garnered attention for their potential in UC management [3,4,5,6,7,8]. Chick early amniotic fluid (ceAF), derived from 7-day-old embryos and rich in nutrients and growth factors such as nerve growth factor (NGF), transforming growth factor-β (TGF-β), vascular endothelial growth factor (VEGF), and insulin-like growth factors (IGF-I and IGF-II), has demonstrated efficacy in various inflammatory models [9,10]. Additionally, ceAF has demonstrated efficacy in inhibiting LPS-induced inflammatory responses in RAW 264.7 macrophages under high-glucose conditions. In a study using a streptozotocin (STZ)-induced diabetic mouse model, ceAF was found to regulate M2 macrophage transformation through the TLR4/NF-κB and Nrf2 signaling pathways, leading to a downregulation of the inflammatory response, alleviation of collagen deposition, and promotion of neovascularization, thereby facilitating diabetic wound healing in C57 mice [11]. Previous studies have also indicated that ceAF improves cardiac function by reducing cardiomyocyte apoptosis, reversing myocardial contractility, and treating ischemic heart injury through the inhibition of NF-κB signaling and the downregulation of inflammatory cytokine expression [12]. The ceAF is abundant in fatty acids (e.g., myristic acid and valeric acid) and amides (e.g., erucamide and nicotinamide), each exhibiting distinct biological activities. Myristic acid has been shown to alleviate 12-O-tetradecanoylphorbol-13-acetate (TPA)-induced skin inflammation and nociception in CD1 mice [13], as well as stimulate appetite responses in human newborns [14]. Valeric acid has been reported to enhance neuroplasticity and cognitive function in mice [15]. Erucamide regulates cholinergic function, alleviating depression and anxiety while improving cognitive abilities [16,17]. Nicotinamide exhibits potent anti-inflammatory and antioxidant effects in neurological and metabolic disorders [18]. Supplementary Table S1 lists the treatment effects for some active ingredients in ceAF. These findings provide a foundation for the natural reparative, anti-inflammatory, and antioxidant effects of ceAF. However, whether ceAF exerts anti-inflammatory effects in UC has not been reported. This study extracted and analyzed ceAF components using mass spectrometry and assessed its anti-inflammatory effects in a dextran sulfate sodium (DSS)-induced UC model in Caco-2 cells and C57BL/6 mice. TNF-α-induced Jurkat cells were employed to elucidate the underlying mechanisms. Our findings suggest that ceAF may offer novel insights into nutritional interventions for inflammatory diseases like UC.

## 2. Materials and Methods

### 2.1. Cell Culture

Caco-2 cells (Wuhan Pricella Biotechnology Co., Ltd., Wuhan, China, Cat NO. CL-0050) were cultured in MEM medium supplemented with 20% FBS and 1% penicillin streptomycin mixture. Jurkat cells (Wuhan Pricella Biotechnology Co., Ltd., Cat NO. CL-0315) were cultured in RPMI-1640 medium supplemented with 10% FBS and 1% penicillin streptomycin mixture. Incubation conditions were as follows: 37 °C, and 5% CO_2_. The culture medium of the cells was changed every other day, and the cells were passaged when they reached 80% to 90% confluence.

### 2.2. Experimental Animals

Eight-week-old male C57/BL6 mice were obtained from Sibeifu Biotechnology Co., Ltd. (Suzhou, Jiangsu, China). The mice were housed at a temperature of 22–26 °C, with relative humidity of 40–70%, and light/dark cycle of 12 h light/12 h dark (8:00 a.m. to 8:00 p.m.), and allowed access to water and food ad libitum. All experiments were conducted in accordance with the relevant regulations of the JiangNan University Laboratory Animal Center for the management and handling of laboratory animals (Approval Number: JN. No20230530c0401220[238]), as well as international guidelines on the ethical use of animals.

### 2.3. Experimental Design

Caco-2 cells were divided into the following groups: control group (cells without any treatment); DSS group (refer to the method of KIM H et al. [19], cultured using 2.5% DSS); and DSS + ceAF group (cultured with appropriate concentrations of ceAF according to the scale of each experiment).

Jurkat cells were divided into the following groups: control group (cells without any treatment); TNF-α group (refer to the method of DALMASSO G et al. [20], cultured using 50 ng/mL TNF-α); and DSS + TNF-α group (cultured with appropriate concentrations of ceAF according to the scale of each experiment). Inhibitor groups were as follows: TNF-α + SB203580 (30 μM) group. Plasmid groups were as follows: TNF-α + pLenti-CMV-LCK group and TNF-α + pLenti-CMV-Control group.

In this study, 8 mice per group were set on the basis of the 3R (reduction, replacement, and refinement) program according to the principles of experimental species design and repeatability. A total of 32 mice were randomly divided Into 4 groups based on their body weight: the control group (pure water ad libitum); the DSS group (free access to 3% DSS for 7 days); DSS + ceAF group (after freely drinking 3% DSS for 7 days, refer to the method of WANG J et al. [12], and perform a gavage of 15 mL/kg ceAF for 10 days); and DSS + Mesalazine group (after freely drinking 3% DSS for 7 days, administered 400 mg/kg Mesalazine by gavage for 10 days).

### 2.4. Reagents and Antibodies

DSS (Cat. NO. 02160110-CF) was from MP Biomedicals Co., Ltd. (Santa Ana, CA, USA); TNF-α was from sinobiological (Beijing, China); SB203580 hydrochloride was from Yuanye Bio-Technology (Shanghai, China); RPMI-1640 medium, MEM medium, Opti-MEM medium, phosphate buffer solution, penicillin streptomycin mixture (100×), trypsin solution (0.25%), and special grade fetal bovine serum (FBS) were products from Gibco (Waltham, MA, USA); urine fecal occult blood test kit, glutathione (GSH) assay kit, and malondialdehyde (MDA) assay kit were obtained from Nanjing Jiancheng Bioengineering Institute (Nanjing, China); superoxide dismutase (SOD) assay kit was from Solarbio (Beijing, China); Cell Counting Kit-8 (CCK8) (Cat. NO. SB-CCK8), BCA protein assay kit (Cat. NO. SB-WB013), RIPA lysate, Total RNA extraction kit (Cat. NO. SB-R001), and cDNA Reverse Transcription Kit (Cat. NO. SB-RT001) were from Share-bio (Shanghai, China); enzyme-linked immunosorbent assay kits for human TNF-α (Cat. NO. EK0525), IL-1β (Cat. NO. EK0392) and IL-10 (Cat. NO. EK0416), and mouse TNF-α (Cat. NO. EK0527) were from Bosterbio (Wuhan, China); mouse IL-1β (Cat. NO. EK201BHS) and IL-10 (Cat. NO. EK201/4) enzyme-linked immunosorbent assay kits were from Multi Sciences (Hangzhou, China); and citric acid antigen repair solution, hematoxylin staining solution, and DAB color reagent were from Servicebio Technology Co., Ltd. (Wuhan, China). Lipofectamine 3000 transfection reagent (Cat. NO. L3000001) was from Thermo Fisher Scientific (Waltham, MA, USA). Other analytical grade reagents, unless otherwise specified, were obtained from a local reagent vendor.

Anti-β-actin (Cat. NO. SB-AB2001) and goat anti-rabbit IgG horseradish peroxidase (HRP)-conjugated secondary antibodies (Cat. NO. SB-AB0101) were from Share-bio (Shanghai, China); and anti-p-LAT (Y220) (Cat. NO. CY5054), anti-total LCK (Cat. NO. CY2972), and anti-total LAT (Cat. NO. AY9064) were obtained from Abways (Shanghai, China); occludin (Cat. NO. R381549), anti-total ZAP70 (Cat. NO. R26132), anti-p-ZAP70 (Tyr319) (Cat. NO. 310211), and anti-p-LCK (Try394) (Cat. NO. 310038) came from ZEN-BIO (Chengdu, China); and ZO-1 (Cat. NO. PB9234) was from Bosterbio (Wuhan, China).

### 2.5. Chick Early Amniotic Fluid (ceAF) Preparation

Fertilized eggs originated from Tiancheng poultry industry (Lianyungang, Jiangsu). Fertilized eggs were incubated at 37 ± 1 °C and 50% humidity. Collect the amniotic fluid from eggs incubated for 6–8 days using a sterile syringe. Collected amniotic fluid was centrifuged at 10,000× *g*/min for 30 min and stored long term at −80 °C. For in vitro experiments, 0.22 μm membranes were used for filtration.

### 2.6. Chemical Composition and Fatty Acid Content Analysis of ceAF

The chemical components of ceAF were analyzed using a Waters ultra-high performance liquid chromatography system (ACQUITY UPLC System) combined with a high-resolution mass spectrometer (TripleTOF 6600, SCIEX, Framingham, MA, USA). To prepare the samples, 200 μL of ceAF was mixed with 400 μL of ice-cold tert-butyl ether and 80 μL of ice-cold methanol, followed by thorough shaking. The mixture was then centrifuged at 3000 rcf for 15 min. The supernatant was freeze-dried, and 200 μL of dichloromethane-methanol solution was added for reconstitution. After another centrifugation at 3000 rcf for 15 min, the supernatant was used for UPLC-HRMS detection. Samples for UPLC-HRMS testing were prepared by mixing 10 to 20 μL of extract from each sample. The chromatographic analysis was performed using an ACQUITY UPLC BEH C18 column (1.7 μm, 100 mm × 2.1 mm, Waters, Milford, MA, USA ). The mobile phase consisted of the following: phase A (acetonitrile: water (6:4) + 10 mmol/L ammonium formate + 0.1% formic acid) and phase B (isopropanol: acetonitrile (9:1) + 10 mmol/L ammonium formate + 0.1% formic acid). The gradient elution was programmed as follows: 0–0.4 min, 30% B; 0.4–1 min, 30–45% B; 1.0–3.5 min, 45–60% B; 3.5–5.0 min, 65–75% B; 5.0–7.0 min, 75–90% B; 7.0–8.5 min, 90–100% B; 8.5–8.6 min, 100% B; 8.6–8.61 min, 100–30% B; and 8.61–10 min, 30% B. The column temperature was maintained at 40 °C, and the flow rate was set at 0.3 mL/min. Each sample underwent both positive and negative mode acquisition. The ion source pressure was maintained at 30 PSI, with auxiliary gas and sheath gas pressures set at 60 PSI. The source temperature was set at 500 °C, and the voltage was adjusted to +5000 V in positive mode and −4500 V in negative mode. Data acquisition was performed in information-dependent acquisition (IDA) mode. In each acquisition cycle, the primary acquisition range was 50–2000 daltons, with a primary acquisition time of 170 ms. The 12 most intense signal ions, with signal accumulation intensity exceeding 100, were selected for secondary fragmentation scanning. Dynamic exclusion was set for 4 s. Instrument accuracy correction was conducted every 20 samples, and QC samples were scanned every 10 samples to correct systematic errors across the entire batch of experiments.

For the detection of fatty acids in ceAF, different concentrations of fatty acid methyl ester mixed standard solution were prepared first, and then ceAF was esterified, extracted, and washed. Following centrifugation, 100 mg of anhydrous sodium sulfate powder was added to remove excess water, then 15 μL of 500 ppm methyl salicylate was added as an internal standard, shaken to mix well, and an appropriate amount of supernatant was pipetted and added to the test vial. Fatty acids in ceAF samples were detected using a Thermo Trace 1300 (Thermo Fisher Scientific, Waltham, MA, USA) gas phase system and a Thermo TSQ 9000 mass spectrometer (Thermo Fisher Scientific, Waltham, MA, USA).

### 2.7. Observation of Signs and Disease Activity Index (DAI) in Mice

Body weight and fecal status were recorded daily during DSS drinking and subsequent gavage administration. According to the method of KIM K M et al. [21], disease activity index was calculated based on body weight, fecal consistency, and the degree of fecal bleeding.

### 2.8. Histomorphopathologic Analysis

A portion of the descending colon of mice was fixed in 4% paraformaldehyde for dehydration and paraffin embedding and subsequently prepared into 4 μm tissue sections. Sections were dewaxed and stained with hematoxylin and eosin dye and Masson dye, respectively, washed, dehydrated, mounted, and then scanned with a Panoramic MIDI scanning whole slide scanner (3DHISTECH Ltd., Budapest, Hungary) and images were viewed at Panoramic Viewer 1.15.4 (3DHISTECH). In addition, histopathological scoring was based on previously developed scoring guidelines [22].

### 2.9. Immunohistochemical Analysis

The 4 µm mouse colon tissue sections were deparaffinized and washed for antigen retrieval, followed by incubation in 3% hydrogen peroxide solution at room temperature in the dark for 25 min, PBS washing (3 times/5 min), and blocking (3% BSA, RT, 30 min). The primary antibody (LCK, 1:1500, p-LCK1:3000, ZAP70, 1:1000, p-ZAP70, 1:1000, LAT, 1:200, p-LAT, 1:200) was incubated overnight at 4 °C, with PBS washing (3 times/5 min); and the secondary antibody was incubated at room temperature for 50 min, with PBS washing (3 times/5 min), DAB staining, and hematoxylin counterstaining of nuclei for 3 min. Sections were scanned by Panoramic MIDI scanning whole slide scanner (3DHISTECH Ltd., Budapest, Hungary). ImageJ software (version 1.52) was used to analyze the positive rate of staining in sections.

### 2.10. Determination of Inflammatory Factors and Antioxidant Indicators

The colon tissue of mice and cell culture supernatants were collected and assayed for TNF-α, IL-1β, and IL-10 according to the instructions of the ELISA kit. The contents of SOD, MDA, and GSH in colon tissue of mice were detected according to the kit instructions.

### 2.11. Cell Viability Assay

Cell viability was determined using the Cytotoxicity Assay CCK-8 according to the kit manufacturer ‘s instructions. Briefly, Caco-2 cells (2 × 10^4^ cells/well) and Jurkat cells (1 × 10^5^ cells/well) were seeded in 96-well plates (Corning, CA, USA) and cultured for 24 h to ensure cell attachment. Caco-2 cells were incubated with MEM (FBS free) medium containing DSS (2.5%) for 24 h to induce inflammation, then cultured with serum-free MEM medium containing ceAF (1% to 20%) for 24 h instead of DSS treatment; the medium of Jurkat cells was replaced with RPMI-1640 (FBS free) containing ceAF (1% to 20%). Absorbance at 450 nm was measured on a microplate reader (PerkinElmer EnSight, Waltham, MA, USA) and cell viability was expressed as the percentage of viable cells relative to untreated controls.

### 2.12. Quantitative RT-PCR (qPCR)

Relative mRNA levels in colon tissues were analyzed by real-time PCR. Total RNA from colon tissue was isolated using a total RNA extraction kit and cDNA was synthesized using a cDNA synthesis kit following the kit’s instructions. Specific primers for *CD3e*, *Itk*, *Cd3d*, *CD8a*, *Lck*, *Lat*, *CD3g*, *CD8b1*, and *GAPDH* were synthesized by Sangon Biotech Co., Ltd. (Shanghai, China) (Supplementary Table S2). Statistical analyses were performed using the 2−ΔΔCT method [23]. *GAPDH* was the internal reference control and three biological replicates were performed for each sample.

### 2.13. RNA-Sequencing Analysis

To determine gene expression changes in UC mice and UC mice after ceAF intervention, colon tissues from mice were snap-frozen in liquid nitrogen for RNA-seq analysis. High-throughput RNA sequencing was performed by Azenta Biotechnology Co., Ltd. (Suzhou, China). Criteria for significantly differentially expressed genes (DEGs) were genes with fold change (FC) ≥ 1 and *p*-value < 0.05 between UC and ceAF intervention groups.

### 2.14. Transfection with Lck Plasmid

Jurkat cells were seeded in 6-well plates (8 × 10^5^ cells/well); the original medium was changed to Opti-MEM when the cells grew to 80% confluence, and Jurkat cells were transfected using Lipofectamine 3000 transfection reagent, as well as pLenti-CMV-LCK group and pLenti-CMV-Control plasmid. After 6 h, Opti-MEM was changed to RPMI-1640 medium. After cells were treated with TNF-α (50 ng/mL) for 24 h, cells were treated with 20% ceAF for 24 h.

### 2.15. Western Blotting Assays

The total protein from mouse tissue, Caco-2, and Jurkat cells was extracted with RIPA lysate containing protease inhibitors and phosphatase inhibitors, and, after quantifying the protein concentration by BCA, sulfate-polyacrylamide gel electrophoresis (SDS-PAGE) was performed using 30 μg of protein, followed by protein transfer, blocking, primary antibodies (ZO-1, 1:5000; Occludin, 1:2000; LCK, 1:2000; p-LCK, 1:2000; ZAP70, 1:1000; p-ZAP70, 1:1000; LAT, 1:1500; p-LAT, 1:2000; β-actin, 1:10,000) undergoing overnight incubation at 4 ° C, washing, and secondary antibodies (1:10,000) for 1 h at room temperature and washing. Optical density was determined by an automated chemiluminescence image analyzer (Tanon, Shanghai, China). Quantitative analysis of bands was performed by ImageJ software.

### 2.16. Statistical Analysis

In this study, experiments were independently repeated no less than three times. The data were analyzed using GraphPad Prism (version 10.0, San Diego, CA, USA) software. The results are presented as mean ± standard deviation (SD) (in vitro experiments) or mean ± standard error of the mean (SEM) (in vivo experiments). Analysis was performed using one-way ANOVA. Post hoc comparisons were conducted using Student–Newman–Keuls test. *p* < 0.05 was considered statistically significant.

## 3. Results

### 3.1. Chemical Composition of ceAF

**Figure 1:**
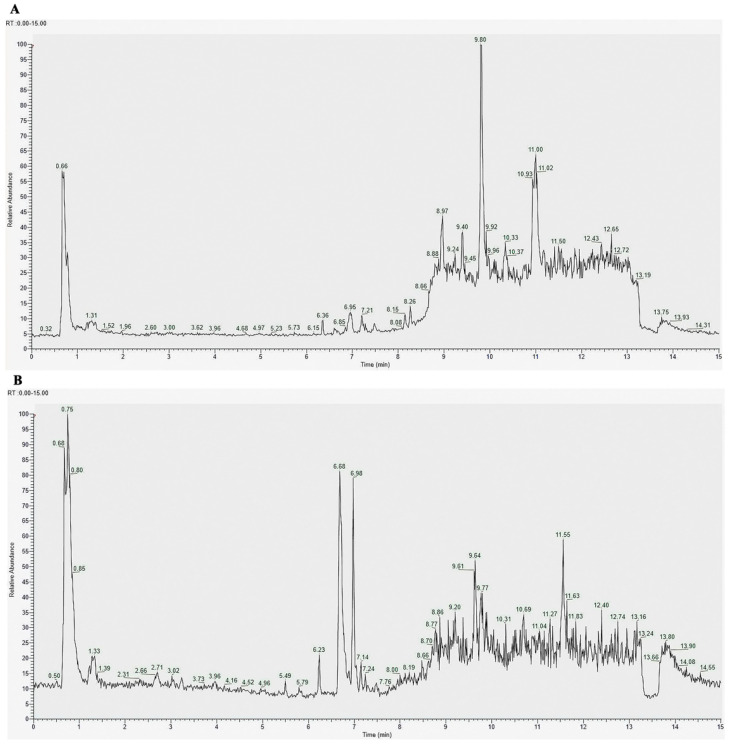
Total ion chromatograms of ceAF: (**A**) base peak chromatogram obtained by negative ion mode; and (**B**) base peak chromatogram obtained by positive ion mode. Abscissa: time; ordinate: relative abundance.

Major compounds in chick early amniotic fluid (ceAF) were analyzed using UPLC-MS, with total ion chromatograms (TICs) presented in Figure 1A (negative ion mode) and Figure 1B (positive ion mode). Through cross-referencing with database entries and existing literature, we identified 249 compounds in ceAF based on their mass spectrometric behavior and retention times. Of these, 119 compounds were detected in negative ion mode and 130 in positive ion mode. The identified compounds predominantly include various fatty acids, aldehydes, amino acids, and amides. A detailed list of these top-ranked compounds is provided in Table 1. Because of the important role played by fatty acids in the intestine, we further examined the fatty acid content in ceAF (Table 2).

### 3.2. Anti-Inflammatory Effect of ceAF in DSS-Induced Caco-2 Cells

**Figure 2:**
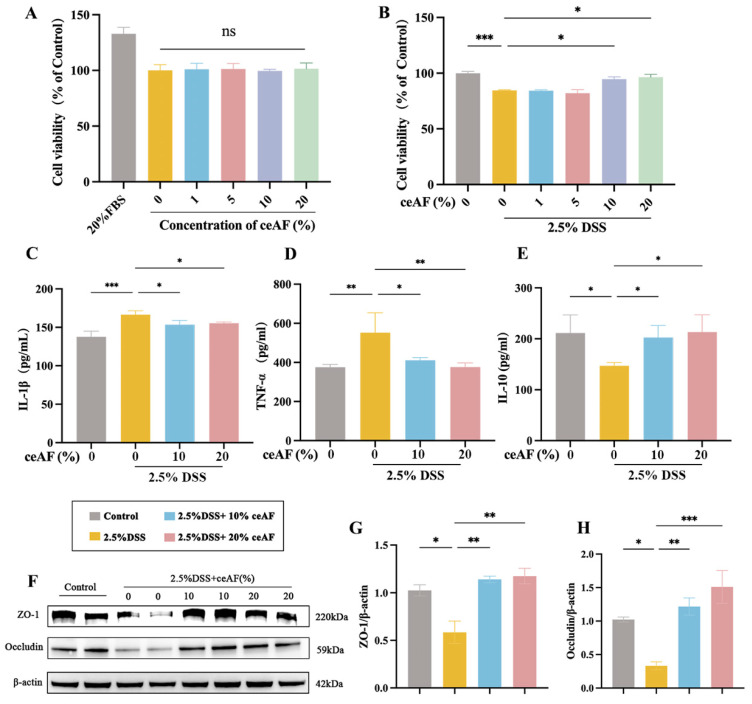
Effect of ceAF on DSS-induced Caco-2 cells. (**A**) Effect of ceAF (1% to 20%) at different concentrations alone for 24 h on the viability of Caco-2 cells. (**B**) Effect of 24 h post-treated with different concentrations of ceAF (1% to 20%) on the viability of Caco-2 cells treated with 2.5% DSS for 24 h. Effect of ceAF (10% and 20%) post-treated on intracellular IL-1β (**C**), TNF-α (**D**), and IL-10 (**E**) levels in Caco-2 cells after 2.5% DSS intervention. (**F**) Western blotting was used to detect the expression of ZO-1 (**G**) and occludin (**H**) after 2.5% DSS and 2.5% DSS + ceAF (10% and 20%) intervention. All data are presented as mean ± SD (*n* = 3); ns, not significant; *, *p* < 0.05; **, *p* < 0.01; ***, *p* < 0.001.

Caco-2 cells were utilized to investigate the protective effects of ceAF against DSS-induced inflammatory responses. Figure 2A illustrates the cytotoxicity assessment, showing that ceAF alone, at concentrations ranging from 1% to 20%, did not affect cell viability after 24 h of treatment. Subsequently, the protective effects of various ceAF concentrations (1% to 20%) on Caco-2 cells exposed to 2.5% DSS were evaluated. Cells were treated with 2.5% DSS for 24 h, followed by ceAF treatment for an additional 24 h. As shown in Figure 2B, ceAF at 10% and 20% concentrations significantly protected Caco-2 cells from DSS-induced cytotoxicity, leading to the selection of these concentrations for further experiments. Tumor necrosis factor-α (TNF-α) and interleukin-1β (IL-1β) are key pro-inflammatory cytokines involved in UC pathogenesis. Exposure to 2.5% DSS markedly upregulated TNF-α (Figure 2C) and IL-1β (Figure 2D), while downregulating the anti-inflammatory cytokine interleukin-10 (IL-10) (Figure 2E). However, ceAF treatment effectively counteracted these effects (Figure 2C–E). Additionally, Western blot analysis confirmed the reparative effects of ceAF on the cell barrier integrity in DSS-induced inflammatory responses in Caco-2 cells. DSS treatment significantly reduced the expression of zonula occludens-1 (ZO-1) (Figure 2F,G) and the tight junction protein occludin (Figure 2F,H). Intervention with 10% and 20% ceAF significantly restored the expression of ZO-1 and occludin in DSS-treated cells (Figure 2F–H). These findings demonstrate the potential of ceAF to mitigate DSS-induced inflammatory responses.

### 3.3. Effect of ceAF on DSS-Induced Pathological Parameters in UC Mice

**Figure 3:**
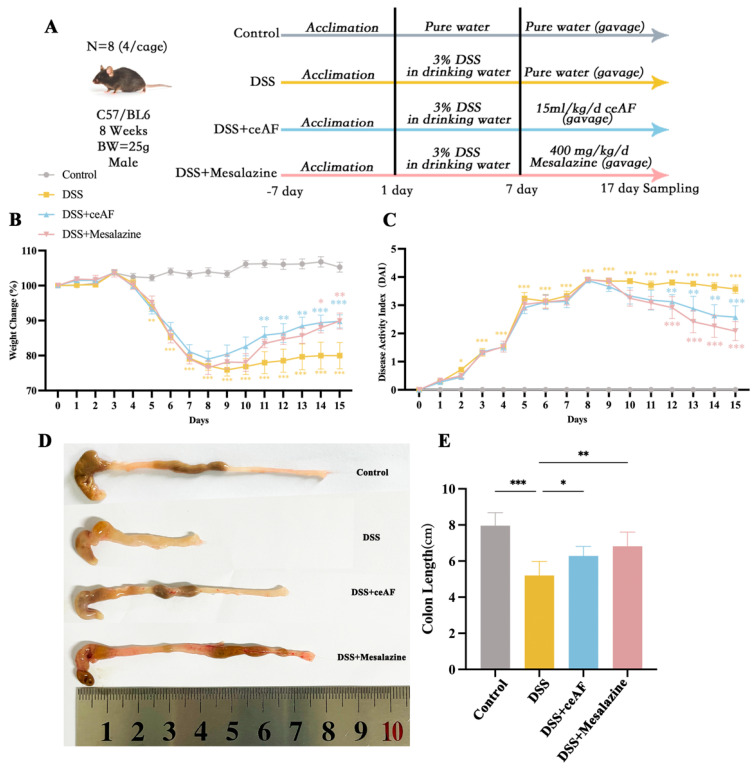
(**A**) Experimental design diagram. Effect of ceAF and mesalazine intervention on DSS-induced body weight (**B**), DAI (**C**), and colon length (**D**,**E**) in C57 mice. All data are presented as mean ± SEM (*n* = 6); ns, not significant; *, *p* < 0.05; **, *p* < 0.01; ***, *p* < 0.001.

**Figure 4:**
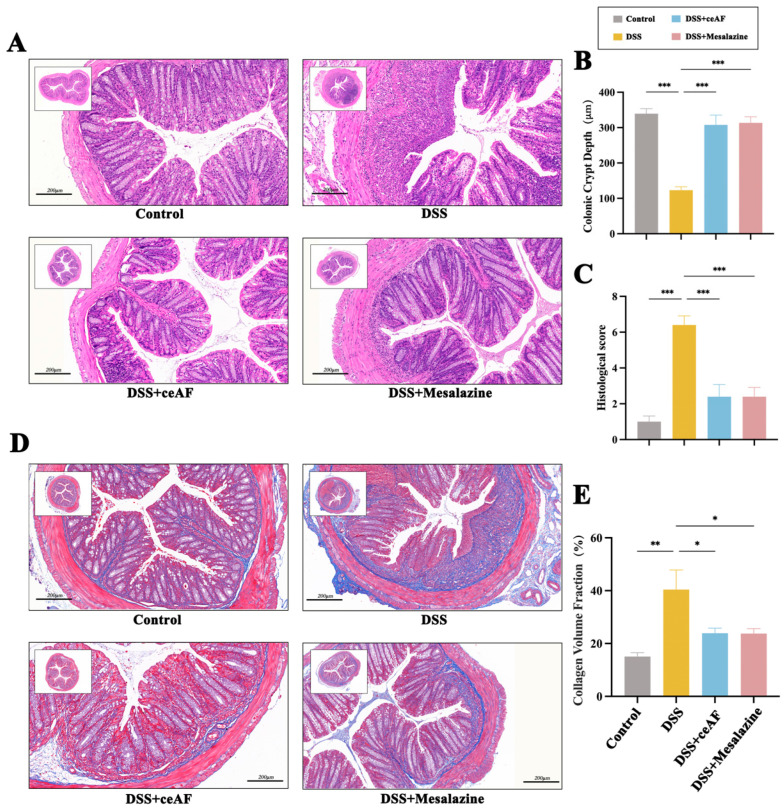
Effect of ceAF and mesalazine intervention on DSS-induced histomorphological changes in colon tissue of C57 mice. Colon tissue sections were stained with (**A**) H&E and (**D**) Masson dyes and observed microscopically. (**B**) Colon histopathology score. (**C**) Colonic crypt depth. (**E**) Collagen volume fraction. Scale bar: 200 μm. All data are presented as mean ± SEM (*n* = 3); *, *p* < 0.05; **, *p* < 0.01; ***, *p* < 0.001.

To further investigate the in vivo effects of ceAF, the experimental design for the animal study is illustrated in Figure 3A. Following induction with 3% DSS, the mice exhibited significant changes in body weight, with the percentage changes in body weight shown in Figure 3B. Compared to the control group, the body weight of the DSS group mice significantly decreased starting on day 5. However, four days after intervention with ceAF and mesalazine (on day 11), the body weight of the ceAF and mesalazine group mice showed a significant upward trend compared to the DSS group. These findings indicate that ceAF effectively alleviated DSS-induced weight loss in UC mice. The Disease Activity Index (DAI) is a comprehensive measure that includes body weight fluctuations, fecal consistency, and rectal bleeding, reflecting the severity of colitis in mice. It is one of the key indicators for evaluating UC. Throughout the experimental period, the DAI index of the control group mice remained at zero. In contrast, the DAI index significantly increased in the DSS group, ceAF group, and mesalazine group mice (Figure 3C). Compared to the DSS group, the DAI index in the ceAF and mesalazine groups began to decrease significantly after five days of treatment (on day 12). The length of the mouse colon is an indicator of inflammation severity. As depicted in Figure 3D,E, compared to the control group, the DSS group mice exhibited significant colon abnormalities, including shortening, congestion, and swelling. Both the ceAF and mesalazine groups significantly improved DSS-induced colon shortening, and colon swelling was also alleviated. To further assess the effect of ceAF on the morphology and structure of colon tissue in UC mice, H&E staining and histopathological scoring were performed on sections of mouse colon tissue. As shown in Figure 4A, a substantial amount of intestinal epithelial cell necrosis, accompanied by extensive inflammatory cell infiltration reaching the submucosa, colonic crypts, and goblet cell loss, was observed in DSS-treated mice compared to control mice. The histopathological scores were also significantly increased (Figure 4B). Treatment with ceAF and mesalazine significantly reduced intestinal epithelial cell necrosis, crypt loss, inflammatory cell infiltration, and histopathological scores compared to the DSS group (Figure 4A,B). The measurement of colonic crypt depth in HE-stained sections revealed a significant reduction in crypt depth in DSS-treated mice compared to the control group (Figure 4C), indicating impaired colonic function. However, a significant increase in colonic crypt depth was observed in the ceAF and mesalazine groups compared to the DSS group (Figure 4C). Intestinal tissue fibrosis is a common complication of UC. As shown in Figure 4D, there was a significant increase in the area of blue collagen fiber deposition in the colonic tissue, with substantial blue deposition observed in the submucosa, muscular layer, and lamina propria of DSS-treated mice compared to control mice. In contrast, the ceAF and mesalazine groups showed a significant reduction in the fibrotic area and collagen volume fraction in intestinal tissue compared to the DSS group (Figure 4E). These results indicate that ceAF significantly ameliorates DSS-induced pathological changes in UC mice, including colon shortening, swelling, inflammatory cell infiltration, and colon fibrosis, exhibiting effects comparable to those of the positive control drug, mesalazine.

### 3.4. ceAF Attenuates DSS-Induced Intestinal Inflammation and Oxidative Damage

**Figure 5:**
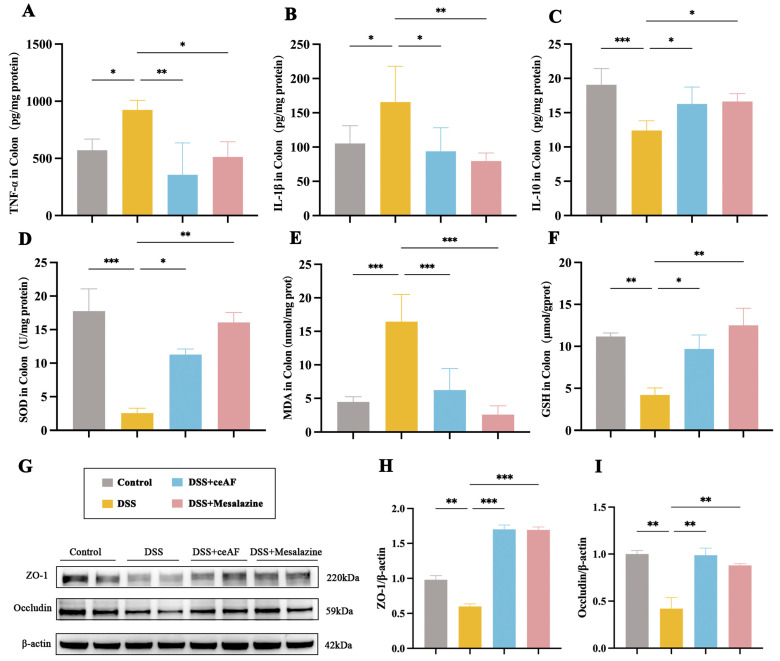
Effect of ceAF and mesalazine intervention on DSS-induced IL-1β (**A**), TNF-α (**B**), IL-10 (**C**), SOD (**D**), MDA (**E**), and GSH (**F**) levels in colon tissue of C57 mice. (**G**) Western blotting was used to detect the expression of ZO-1 (**H**) and occludin (**I**) in colon tissue after ceAF and mesalazine intervention; all data were expressed as mean ± SEM (*n* = 6); *, *p* < 0.05; **, *p* < 0.01; ***, *p* < 0.001.

To investigate the anti-inflammatory protective effects of ceAF in DSS-induced UC mice, we measured the levels of the pro-inflammatory factors TNF-α and IL-1β, as well as the anti-inflammatory factor IL-10, in the colon tissue. Compared to the control group, the DSS group exhibited a significant increase in the levels of TNF-α and IL-1β in colon tissues (Figure 5A,B), while the levels of IL-10 were significantly decreased (Figure 5C). In contrast, the ceAF-treated group showed a significant reduction in the levels of TNF-α and IL-1β compared to the DSS group, as well as a notable increase in IL-10 levels (Figure 5A–C). Several indicators of antioxidants, including SOD, MDA, and GSH, exhibited significant alterations following the ceAF intervention. The ceAF treatment markedly enhanced superoxide dismutase (SOD) and glutathione (GSH) activities (Figure 5D,F) while concurrently reducing malondialdehyde (MDA) levels (Figure 5E) in comparison to the DSS group, indicating that the ceAF intervention mitigated the oxidative damage induced by DSS.

Furthermore, the repair effects of ceAF on the intestinal barrier damage caused by DSS-induced inflammatory responses in UC mice were assessed using Western blotting. DSS treatment significantly reduced the expression of the tight junction proteins ZO-1 and occludin (Figure 5G–I). However, ceAF treatment notably improved the downregulation of ZO-1 and occludin induced by DSS in the colon tissue (Figure 5G–I).

### 3.5. General Characteristics of ceAF vs. DSS Transcriptomic Analysis

**Figure 6:**
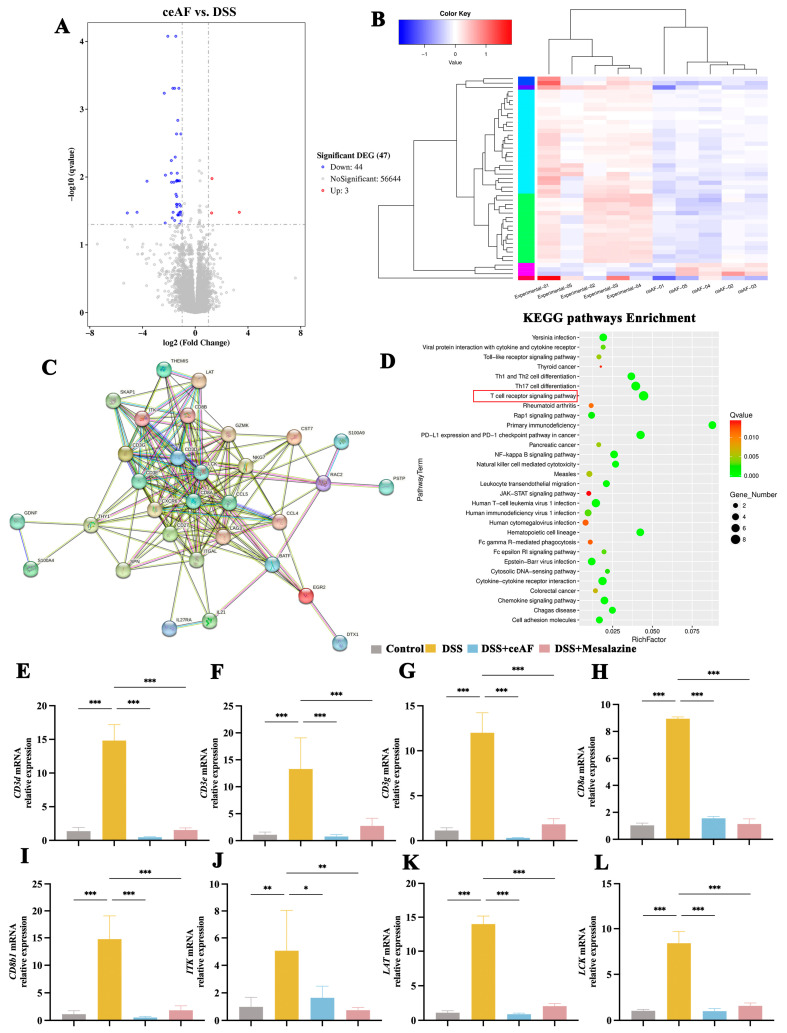
Transcriptome analysis of colon tissue between ceAF and DSS groups. (**A**) Volcano plot of differentially expressed genes (DEGs) between ceAF and DSS groups. (**B**) Heatmap of DEGs between ceAF and DSS groups. Red and blue represent up- and downregulated genes, respectively. (**C**) Predicted protein interaction (PPI) networks based on DEGs. (**D**) KEGG pathways predicted based on DEGs. Real-time PCR was used to detect the mRNA levels of *CD3d* (**E**), Cd3E (**F**), CD3g (**G**), *CD8a* (**H**), *CD8b1* (**I**), *Itk* (**J**), *Lat* (**K**), and *Lck* (**L**) in DSS-induced colon tissue of C57 mice from ceAF and mesalazine intervened. Red box indicates signaling pathways focused according to *p*-value and number of genes. All data were expressed as mean ± SEM (*n* = 3); *, *p* < 0.05; **, *p* < 0.01; ***, *p* < 0.001.

To further elucidate the mechanism by which ceAF exerts its anti-inflammatory protective effects, a transcriptomic analysis was conducted on colon tissue from UC mice with and without ceAF intervention. The RNA sequencing data revealed that ceAF upregulated 3 genes and downregulated 44 genes compared to the DSS group (Figure 6A,B). Figure 6C illustrates the interaction between these differentially expressed genes (DEGs). A KEGG pathway enrichment analysis of DEGs revealed that the T-cell receptor (TCR) signaling pathway was the most significantly altered by the ceAF intervention (Figure 6D). Within this pathway, eight DEGs were identified: *CD3e*, *Itk*, *Cd3d*, *CD8a*, *Lck*, *Lat*, *CD3g*, and *CD8b1*. Figure 6E–L illustrate the mRNA expression levels of these genes in colon tissue from DSS-induced UC mice following ceAF intervention. In alignment with the sequencing data, DSS treatment significantly upregulated the mRNA levels of these genes, while the ceAF intervention mitigated this upregulation, demonstrating effects comparable to those of the positive control drug, mesalazine.

### 3.6. ceAF Alleviates DSS-Induced Hyperactivation of the TCR Signaling Pathway in Colon Tissue of UC Mice

**Figure 7:**
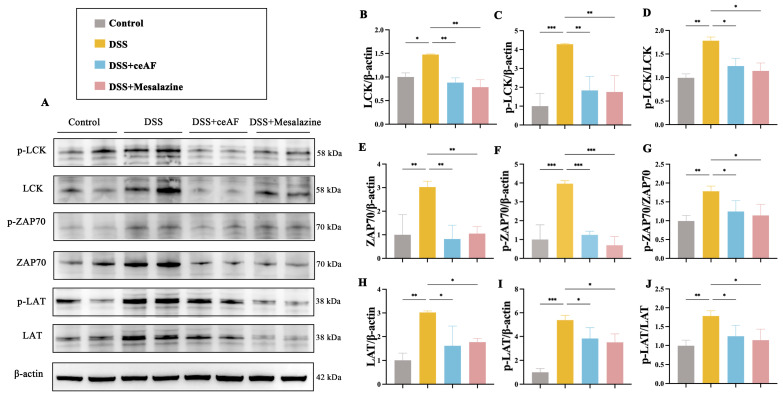
Effect of ceAF and mesalazine intervention on DSS-induced TCR signaling in colon tissue of C57 mice. (**A**) Western blotting was used to detect the expression of LCK (**B**), p-LCK (**C**), p-LCK/LCK (**D**), ZAP70 (**E**), p-ZAP70 (**F**), p-ZAP70/ZAP70 (**G**), LAT (**H**), p-LAT (**I**), and p-LAT/LAT (**J**) in DSS-induced colon tissue of C57 mice from ceAF and mesalazine intervened. All data were expressed as mean ± SEM (*n* = 3); *, *p* < 0.05; **, *p* < 0.01; ***, *p* < 0.001.

**Figure 8:**
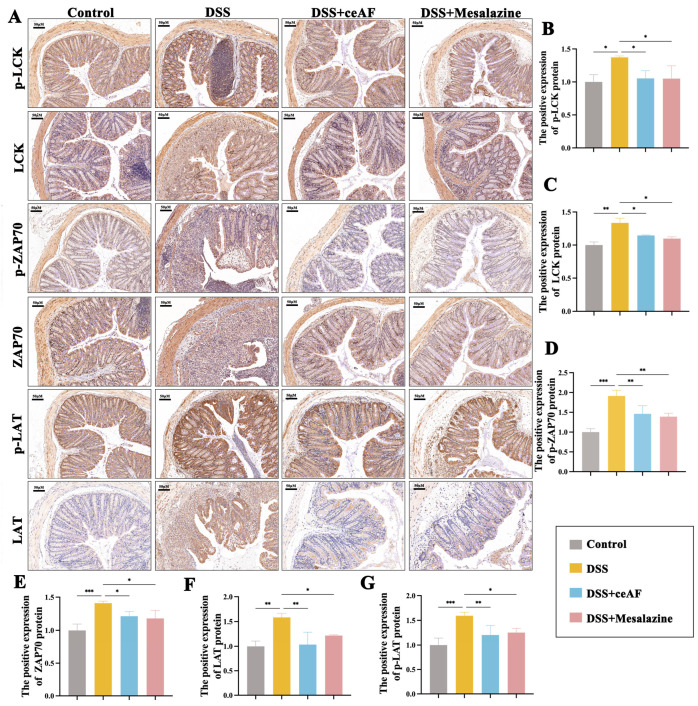
Effect of ceAF and mesalazine intervention on DSS-induced TCR signaling in colon tissue of C57 mice. (**A**) Immunohistochemical staining was used to detect the expression of p-LCK (**B**), LCK (**C**), p-ZAP70 (**D**), ZAP70 (**E**), LAT (**F**), and p-LAT (**G**) in DSS-induced colon tissue of C57 mice from ceAF and mesalazine intervened. All data were expressed as mean ± SEM (*n* = 3); *, *p* < 0.05; **, *p* < 0.01; ***, *p* < 0.001.

Given the critical role of the TCR signaling pathway in inflammation, ceAF may mitigate DSS-induced UC by inhibiting the overactivation of this pathway. To test this hypothesis, we examined colon tissue from DSS-induced UC mice treated with ceAF, focusing on LCK, ZAP70, and LAT—core proteins regulating TCR signaling—and their phosphorylation levels. Western blotting results demonstrated that, compared to the control group, DSS treatment significantly upregulated the expression and phosphorylation of LCK, ZAP70, and LAT. However, this upregulation was inhibited by ceAF and mesalazine (Figure 7A–J). Immunohistochemical staining further confirmed that ceAF inhibited the DSS-induced activation of LCK, ZAP70, and LAT in the TCR signaling pathway, concurrently downregulating their phosphorylation levels (Figure 8A–G).

### 3.7. ceAF Inhibits the TCR Signaling Pathway in Jurkat Cells via the LCK/ZAP70/LAT Axis

**Figure 9:**
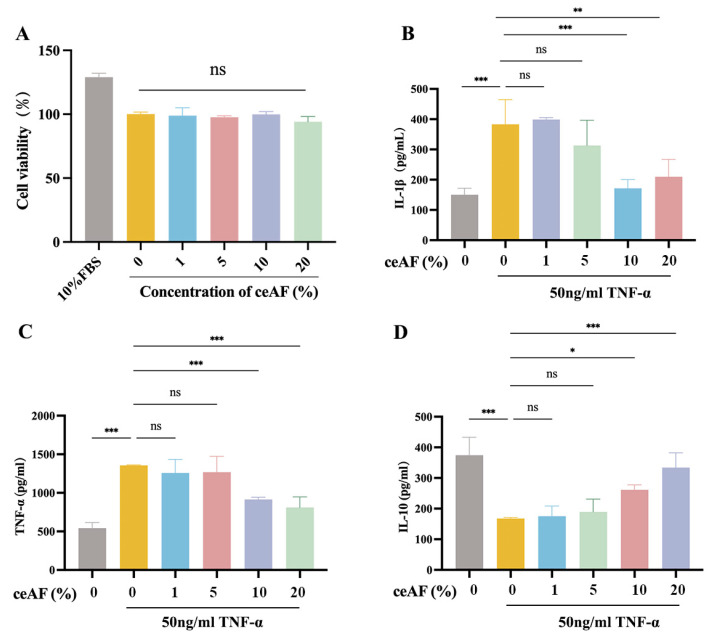
Effect of ceAF on TNF-α-induced Jurkat cells. (**A**) Effect of ceAF (1% to 20%) at different concentrations alone for 24 h on the viability of Jurkat cells. Effect of ceAF (1% to 20%) post-treated on intracellular IL-1β (**B**), TNF-α (**C**), and IL-10 (**D**) levels in Jurkat cells after 50 ng/mL TNF-α intervention. All data are presented as mean ± SD (*n* = 3); ns, not significant; *, *p* < 0.05; **, *p* < 0.01; ***, *p* < 0.001.

**Figure 10:**
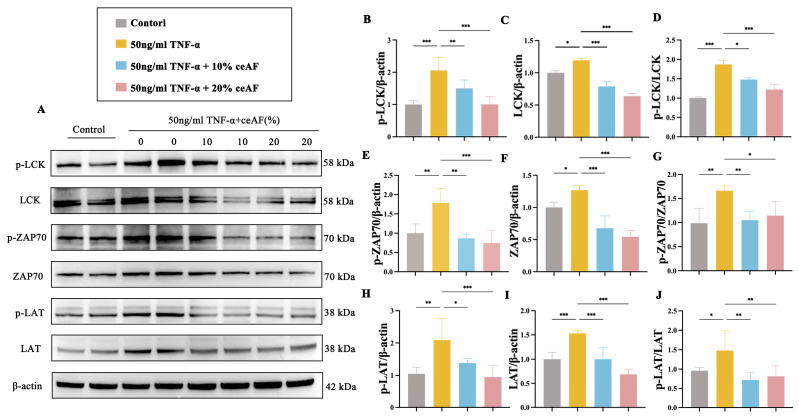
Effect of ceAF intervention on TNF-α-induced TCR signaling pathway in Jurkat cells. (**A**) Western blotting was used to detect p-LCK (**B**), LCK (**C**), p-LCK/LCK (**D**), p-ZAP70 (**E**), ZAP70 (**F**), p-ZAP70/ZAP70 (**G**), p-LAT (**H**), LAT (**I**), and p-LAT/LAT (**J**) expression in Jurkat cells after intervention with single TNF-α or combined ceAF intervention. All data were expressed as mean ± SD (*n* = 3); *, *p* < 0.05; **, *p* < 0.01; ***, *p* < 0.001.

**Figure 11:**
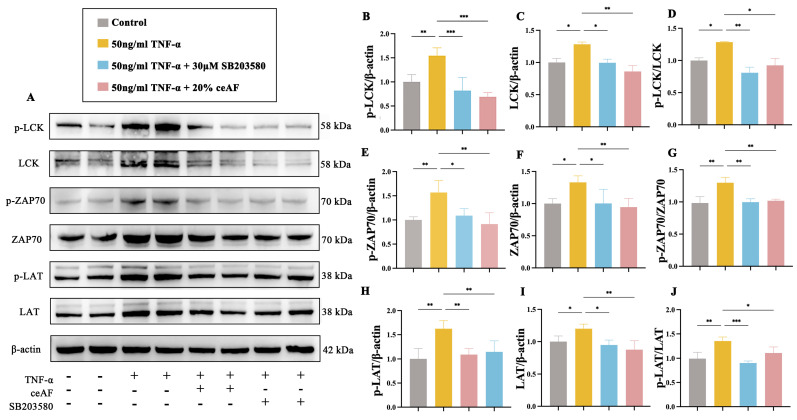
Effect of ceAF and LCK inhibitor SB203580 intervention on TNF-α-induced TCR signaling pathway in Jurkat cells. (**A**) Western blotting was used to detect the expression of p-LCK (**B**), LCK (**C**), p-LCK/LCK (**D**), p-ZAP70 (**E**), ZAP70 (**F**), p-ZAP70/ZAP70 (**G**), p-LAT (**H**), LAT (**I**), and p-LAT/LAT (**J**) in Jurkat cells treated with TNF-α alone or in combination with ceAF or SB203580. All data were expressed as mean ± SD (*n* = 3); *, *p* < 0.05; **, *p* < 0.01; ***, *p* < 0.001.

The human T lymphocyte cell line Jurkat was used to further explore the molecular mechanisms by which ceAF regulates the LCK/ZAP70/LAT-mediated TCR signaling pathway. Initially, the cytotoxic effects of ceAF on Jurkat cells were assessed, revealing that treatment with ceAF alone (1% to 20%) for 24 h did not impact cell viability (Figure 9A). Subsequently, the effects of different ceAF concentrations (1% to 20%) on the TNF-α-induced inflammatory response in Jurkat cells were evaluated. Jurkat cells were first treated with 50 ng/mL TNF-α for 24 h, followed by ceAF treatment for an additional 24 h. The results showed that TNF-α significantly upregulated TNF-α (Figure 9B) and IL-1β (Figure 9C), while downregulating the anti-inflammatory cytokine IL-10 (Figure 9D) compared to the control group. Both 10% and 20% ceAF effectively counteracted these effects (Figure 9B–D). Subsequently, the impact of ceAF on TCR signaling pathways in TNF-α-induced Jurkat cells was examined. The findings indicated that TNF-α significantly upregulated the expression and phosphorylation levels of LCK, ZAP70, and LAT in Jurkat cells compared to the control group, with these effects being inhibited by ceAF (Figure 10A–J). SB203580 is a selective inhibitor that inhibits LCK activity in cells. TNF-α-induced Jurkat cells were treated with ceAF or SB20358 to verify whether ceAF had similar effects to the inhibitor SB203580. The results of Western blotting showed that ceAF was consistent with the effect of SB203580, an LCK inhibitor, and was able to inhibit the expression of LCK, ZAP70, LAT, and their phosphorylation levels in Jurkat cells upregulated by TNF-α (Figure 11A–J).

**Figure 12:**
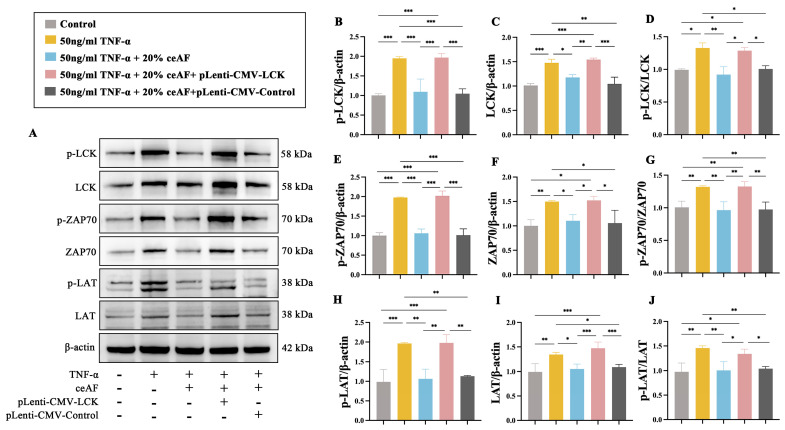
The effect of ceAF intervention on TNF-α-induced TCR signaling pathway in Jurkat cells was examined after overexpression of LCK. (**A**) Western blotting was used to detect the expression of p-LCK (**B**), LCK (**C**), p-LCK/LCK (**D**), p-ZAP70 (**E**), ZAP70 (**F**), p-ZAP70/ZAP70 (**G**), p-LAT (**H**), LAT (**I**), and p-LAT/LAT (**J**) in Jurkat cells after TNF-α, TNF-α + ceAF + pLenti-CMV-LCK, and TNF-α + ceAF + pLenti-CMV-Control intervention. All data were expressed as mean ± SD (*n* = 3); *, *p* < 0.05; **, *p* < 0.01; ***, *p* < 0.001.

To investigate whether ceAF exerts its anti-inflammatory effects through the regulation of the LCK/ZAP70/LAT axis, LCK was overexpressed in Jurkat cells. Compared to the control group, TNF-α significantly upregulated the expression of LCK, ZAP70, LAT, and their phosphorylation levels, which were downregulated in the ceAF-treated group (Figure 12A–J). While the overexpressed LCK successfully induced significant LCK expression, ceAF did not reduce this expression (Figure 12A,C). Moreover, ceAF was unable to decrease the expression of ZAP70, LAT, or their phosphorylation levels in the pLenti-CMV-LCK group (Figure 12A,E–J). These findings collectively suggest that ceAF exerts its anti-inflammatory protective effects by modulating the LCK/ZAP70/LAT-mediated TCR signaling pathway.

## 4. Discussion

The increasing prevalence of UC is becoming a major global health concern, largely driven by rapid lifestyle changes and heightened mental stress [24]. Although the pathogenesis of UC is highly complex, there is a growing body of evidence that highlights the importance of targeting anti-inflammatory pathways to effectively manage the condition [25,26,27,28,29]. Given UC’s prominence as a digestive disorder, the development of FFs as adjuncts to nutritional interventions holds considerable promise. Recent studies on protective formulations derived from traditional Chinese medicine and natural ingredients have provided new perspectives for the development of nutritional intervention and FFs. Prior research on ceAF has shown that it possesses several biological activities, including anti-inflammatory, antioxidant, and anti-myocardial infarction properties. For instance, ceAF has been shown to promote wound healing by regulating M2 macrophage transformation and reducing inflammatory cell infiltration [11,30]. In this study, ceAF demonstrated anti-inflammatory and reparative effects against UC using a DSS-induced model, both in vitro with Caco-2 cells and in vivo with C57BL/6 mice. Specifically, ceAF reduced DSS-induced levels of pro-inflammatory cytokines TNF-α and IL-1β, while increasing the expression of the anti-inflammatory cytokine IL-10. Furthermore, ceAF elevated the levels of SOD and GSH, which are indicators of antioxidant activity, while concurrently decreasing the MDA content. This suggests that ceAF may mitigate oxidative stress injury induced by DSS. These findings suggest that ceAF could have significant protective effects against UC.

Further analysis of ceAF compounds using UPLC-MS identified 249 components, including nucleosides and fatty acids, which were present in different ionization modes. Notably, fatty acids like palmitic acid, stearic acid, and valeric acid, which were not identified in previous studies [30], were found to be abundant. Given that fatty acids play crucial roles in energy metabolism and inflammation, their presence could be a major contributor to ceAF’s anti-inflammatory properties. This is supported by clinical and animal model studies [4,31,32,33,34,35], which suggest that fatty acids improve the quality of life in patients with Crohn’s disease and UC by mitigating inflammation and oxidative stress. Exosomes (EXOs) have been identified in mammalian amniotic fluid and are known to play a critical role in cell-to-cell communication [36,37]. Amniotic-fluid-derived exosomes (AF-EXOs) have been implicated in immune regulation [38] and have been shown to mitigate intestinal injury in experimental models [39,40]. Acting as carriers of bioactive molecules, AF-EXOs are encapsulated by phospholipid bilayers, which protect their cargo from degradation in the gastrointestinal tract and facilitate absorption into deeper regions of the gut [36,41]. This protective mechanism may partly explain the biological activity of ceAF, as fatty acids, proteins, and nucleosides undergo partial degradation during digestion and absorption. Our findings reveal that ceAF is rich in fatty acids, many of which enter systemic circulation through the portal vein or lymphatic system. Consequently, it is plausible that ceAF exerts its anti-inflammatory effects through multiple pathways and targets, rather than through localized actions alone.

UC’s hallmark symptoms, such as intestinal necrosis and barrier impairment, are primarily driven by chronic inflammation and oxidative stress. This damage to the intestinal barrier reduces the expression of tight junction proteins, exacerbating disease progression and potentially leading to colonic fibrosis [42,43,44,45,46]. A recent study showed that fibroblast markers were significantly elevated in the blood of UC patients and were associated with immune activation status [47]. This suggests that chronic inflammation and oxidative-stress-induced intestinal barrier damage and fibrosis are important links in the development of UC. In our study, ceAF at concentrations of 10% and 20% significantly protected Caco-2 cells from DSS-induced cytotoxicity and improved cell viability. Additionally, ceAF upregulated the tight junction proteins ZO-1 and occludin in both Caco-2 cells and DSS-induced C57BL/6 mice, which indicates its ability to reinforce the intestinal barrier. Furthermore, ceAF improved colonic shortening, crypt depth, and fibrosis in DSS-induced UC mice, demonstrating an efficacy comparable to mesalazine [48].

The role of intestinal intraepithelial lymphocytes (IELs) in maintaining immune homeostasis is well-established, and their dysregulation has been linked to UC pathogenesis [49]. TCR signaling, particularly through the Ras-MAPK and NF-κB pathways, plays a pivotal role in T-cell activation and immune regulation [50,51,52,53,54,55]. Previous studies have implicated the TCR signaling pathway in UC development, especially with the overexpression of the IRF5 protein exacerbating disease severity [56,57]. Consistent with these findings, our KEGG enrichment analysis revealed that the TCR signaling pathway is a critical target of ceAF in ameliorating UC. ceAF was found to inhibit key genes in this pathway, including *CD3e*, *Itk*, *CD8a*, and *Lck* in DSS-treated mice.

Lck, a lymphocyte-specific protein tyrosine kinase, is essential for TCR signaling by phosphorylating downstream molecules like ZAP70 and LAT [58,59,60,61,62]. The overexpression of Lck has been associated with the development of UC, and its inhibition has been shown to reduce T-cell activation and alleviate UC symptoms [63,64,65,66]. This suggests that LCK may be involved in UC development by regulating downstream ZAP70 and LAT. In addition, some studies have reported the role of LCK in diabetes, indicating that the interaction of protein tyrosine phosphatase with Src kinase Lck is associated with type I diabetes [67,68], and the inhibition of Lck can inhibit T-cell activation to treat type I diabetes [69], whereas ceAF has already been shown to ameliorate inflammatory responses in STZ-induced diabetic mice and to promote healing of diabetic wounds in mice [11]. These studies provide evidence that ceAF may exert anti-inflammatory effects through the LCK/ZAP70/LAT pathway. Consistent with this, in this study, our results demonstrated that DSS significantly upregulated the expression and phosphorylation levels of LCK, ZAP70, and LAT, a phenomenon that was inhibited by ceAF or mesalazine. These findings were further validated by immunohistochemical staining, confirming the regulatory effect of ceAF on the TCR signaling pathway in UC. These findings collectively indicate that ceAF exerts anti-inflammatory effects through the inhibition of TCR signaling pathway activation. To further explore the molecular mechanisms by which ceAF regulates the LCK/ZAP70/LAT-mediated TCR signaling pathway, we employed a TNF-α-induced inflammatory model in Jurkat cells in vitro. Similar to the observations made in Caco-2 cells, various concentrations of ceAF (ranging from 1% to 20%) were not cytotoxic to Jurkat cells. Notably, treatment with 10% and 20% ceAF significantly reduced TNF-α-induced inflammatory responses in Jurkat cells. Specifically, ceAF downregulated the expression levels of the pro-inflammatory cytokines TNF-α and IL-1β, while upregulating the anti-inflammatory cytokine IL-10. Additionally, a Western blot analysis revealed that both 10% and 20% ceAF inhibited the TNF-α-induced expression and phosphorylation levels of LCK, ZAP70, and LAT in Jurkat cells. Interestingly, the effect of 20% ceAF was comparable to that of SB203580, a selective inhibitor of LCK. Furthermore, the protective effect of ceAF was abrogated following the overexpression of LCK. This is similar to previous results that show that the overexpression of LCK leads to the development of UC. These common results suggest that ceAF exerts anti-inflammatory protective effects by regulating TCR signaling pathways through the LCK regulation of its downstream ZAP70 and LAT expression.

In conclusion, this study provides the first evidence that ceAF can mitigate ulcerative colitis (UC) and TNF-α-induced inflammatory responses by inhibiting the LCK/ZAP70/LAT signaling pathway, thereby reducing TCR signaling hyperactivation. Additionally, ceAF improved several pathological features of UC, including cellular necrosis, colonic shortening, and fibrosis. CeAF’s non-toxic and readily available nature suggests its potential as a nutritional intervention or functional food for managing UC. To obtain more adequate conclusions, future studies may examine alternative modes of administration (e.g., enema or intraperitoneal injection) and analyze data on ceAF in zygotes from different sources and breeds. In addition, we should investigate the clinical correlation between active components of ceAF (such as fatty acids, nucleosides, and amides) and UC patients and explore other formulations of ceAF (e.g., lyophilized powder or concentrate) to enhance human utilization. Finally, we should focus on isolating and purifying the bioactive components of ceAF, including ceAF-EXOs, to further explore its therapeutic potential, particularly in immune-mediated diseases like UC.

## Figures and Tables

**Table 1:** Compounds in ceAF detected by UPLC-MS.

**Negative Ion Mode**					
**No.**	**Compound Name**	**Formula**	***m*** **/*z***	**RT [min]**	**Group Area**
1	Palmitic acid	C16 H32 O2	255.23287	9.813	4,357,510,384
2	Stearic acid	C18 H36 O2	283.26453	10.958	4,061,435,490
3	Valeric acid	C5 H10 O2	101.06094	13.288	2,520,589,319
4	Myristic acid	C14 H28 O2	227.20191	8.96	870,899,862.9
5	dl-Lactic acid	C3 H6 O3	89.02454	0.791	358,035,153.6
6	Kojic acid	C6 H6 O4	141.01717	0.664	348,432,111.8
7	Caprylic acid	C8 H16 O2	143.10794	13.207	244,523,673.5
8	2,6-di-tert-Butylphenol	C14 H22 O	205.15992	8.259	177,305,015.2
9	Oleic acid	C18 H34 O2	281.2489	9.978	155,150,822.5
10	Pentadecanoic acid	C15 H30 O2	241.21766	9.384	149,309,887.4
11	Lauric acid	C12 H24 O2	199.17056	8.143	148,878,532.1
12	Uric acid	C5 H4 N4 O3	167.02128	1.196	130,110,907.9
13	Palmitoleic acid	C16 H30 O2	253.21766	9.193	100,995,495.3
14	4-Dodecylbenzenesulfonic acid	C18 H30 O3 S	325.18482	10.975	94,350,125.41
15	3-Hydroxybutyric acid	C4 H8 O3	103.04017	1.078	78,136,007.21
**Positive Ion Mode**					
**No** **.**	**Compound Name**	**Formula**	***m*** **/*z***	**RT [min]**	**Group Area**
1	2-Amino-1,3,4-octadecanetriol	C18 H39 N O3	318.30018	6.677	1,609,178,255
2	Erucamide	C22 H43 N O	338.34161	11.545	1,603,181,304
3	Choline	C5 H13 N O	104.10702	0.754	1,184,765,704
4	Hexamethylenetetramine	C6 H12 N4	141.1135	13.246	851,641,374
5	Granisetron	C18 H24 N4 O	313.19831	8.857	782,405,096.8
6	Bis(4-ethylbenzylidene)sorbitol	C24 H30 O6	415.21155	6.97	593,893,594.6
7	Nicotinamide	C6 H6 N2 O	123.05532	2.374	444,547,455.8
8	l-Phenylalanine	C9 H11 N O2	166.08634	2.687	295,842,507.5
9	δ-Valerolactam	C5 H9 N O	100.07577	2.922	288,263,436.4
10	*N*-cyclohexyl-1-methyl-5-(1*H*-pyrrol-1-yl)-1*H*-pyrazole-4-carboxamide	C15 H20 N4 O	273.16737	13.23	251,908,905
11	Indan-1-one 1-(4,5-dihydro-1*H*-imidazol-2-yl)hydrazone	C12 H14 N4	215.12525	8.84	219,406,038
12	Betaine	C5 H11 N O2	118.08628	0.735	210,445,968.9
13	Hypoxanthine	C5 H4 N4 O	137.04589	2.071	186,643,512.7
14	l-Norleucine	C6 H13 N O2	132.10202	1.328	178,446,405.3
15	2-[(3*S*)-1-Isopropyl-3-pyrrolidinyl]-1H-benzimidazole-5-carbonitrile	C15 H18 N4	255.15664	8.865	157,960,783

**Table 2:** Content of fatty acids in ceAF.

No.	Fatty Acid	Content (μg/mL)	No.	Fatty Acid	Content (μg/mL)
1	Methyl Oleate	43.491	20	Methyl Elaidate	0.388
2	Methyl Palmitate	32.613	21	Methyl 10-Transnonadecenoate	0.323
3	Methyl Linoelaidate	11.532	22	Methyl 11-Eicosenoate	0.323
4	Methyl Stearate	11.113	23	Methyl Petroselaidate	0.32
5	Methyl Palmitoleate	3.74	24	Methyl Transvaccenate	0.314
6	Methyl Transvaccenate	2.551	25	Methyl Nervonoate	0.3
7	Methyl Petroselaidate	2.249	26	Methyl Erucate	0.287
8	Methyl 10-Heptadecenoate	1.495	27	Methyl Heptadecanoate	0.197
9	Methyl Arachidonate	1.461	28	Methyl Brassidate	0.177
10	Methyl Myristoleate	1.178	29	Methyl Alpha Linolenate	0.15
11	Methyl Myristelaidate	1.061	30	Methyl Pentadecanoate	0.098
12	Methyl 10-Transpentadecenoate	0.804	31	Methyl Lignocerate	0.035
13	Methyl 10-Pentadecenoate	0.556	32	Methyl Linoelaidate	0.032
14	Methyl 10-Transsheptadecenoate	0.556	33	Methyl Laurate	0.02
15	Methyl Trans 11-Eicosenoate	0.45	34	Methyl Docosadienoate	0.014
16	Methyl Docosahexaenoate	0.448	35	Methyl Undecanoate	0.005
17	Methyl Docosapentaenoate	0.416	36	Methyl Caprate	0.002
18	Methyl Palmitelaidate	0.412	37	Methyl Docosatetraenoate	0.002
19	Methyl Myristate	0.404			

## Data Availability

Datasets generated and analyzed during the current study have been uploaded into the GEO database as specified (accession number: GSE280748, token number: oxuxiucgpfohtod).

## Supplementary Materials

The following supporting information can be downloaded at: https://www.mdpi.com/article/10.3390/antiox14010051/s1, Table S1. Therapeutic effect of active ingredient in ceAF [13,14,15,16,17,18,70,71,72,73,74]. Table S2. Primer sequences used in quantitative RT-PCR assays.
