# Supplementary material for: Chick Early Amniotic Fluid Alleviates Dextran-Sulfate-Sodium-Induced Colitis in Mice via T-Cell Receptor Pathway
Source: Antioxidants (Basel). 2025 Jan 4;14(1):51. doi: 10.3390/antiox14010051 (PMC11762673; doi:10.3390/antiox14010051)
Supplement: Supplementary file 1 [file antioxidants-14-00051-s001.zip › Supplementary Table S1 .pdf]

Supplementary Table S1. Therapeutic effect of active ingredient in ceAF

No.	Ingredients	Therapeutic utility	References
1	Myristic acid	It can reduce 12-O-tetradecanoylphorbol-13-acetate (TPA) -induced skin inflammation and nociception in CD1 mice	[1,2]
2	Valeric acid	It can improve neuroplasticity and cognitive function in mice	[3]
3	Kojic acid	Inhibition of aging in human corneal endothelial cells via NF-κB and p21 signaling pathways	[4]
4	Caprylic acid	Enhancing fat metabolism through inhibition of balanced nucleoside transporter 1	[5]
5	Oleic acid	Direct inhibition of tumor growth by unfolded protein response	[6]
6	Erucamide	Can modulate cholinergic function, improving depression and anxiety and cognitive performance	[7,8]
7	Nicotinamide	Good anti-inflammatory and antioxidant effects in nervous system and metabolic diseases	[9]
8	Choline	Enhancing lifelong memory in rat pups	[10]
9	Betaine	It acts via ligand-gated ion channels to control behavioral states	[11]
10	Hypoxanthine	It can enhance ATP production, thereby reducing radiation damage in vascular endothelial cells and mouse skin	[12]

References.

1. Alonso-Castro, A.J.; Serrano-Vega, R.; Pérez Gutiérrez, S.; Isiordia-Espinoza, M.A.; Solorio-Alvarado, C.R. Myristic acid reduces skin inflammation and nociception. *J Food Biochem* **2022**, *46*, e14013, doi:10.1111/jfbc.14013.
2. Gutiérrez-García, A.G.; Contreras, C.M.; Díaz-Marte, C. Myristic acid in amniotic fluid produces appetitive responses in human newborns. *Early Hum Dev* **2017**, *115*, 32-37, doi:10.1016/j.earlhumdev.2017.08.009.
3. Lai, Z.; Shan, W.; Li, J.; Min, J.; Zeng, X.; Zuo, Z. Appropriate exercise level attenuates gut dysbiosis and valeric acid increase to improve neuroplasticity and cognitive function after surgery in mice. *Mol Psychiatry* **2021**, *26*, 7167-7187, doi:10.1038/s41380-021-01291-y.
4. Wei, X.; Luo, D.; Yan, Y.; Yu, H.; Sun, L.; Wang, C.; Song, F.; Ge, H.; Qian, H.; Li, X.; et al. Kojic acid inhibits senescence of human corneal endothelial cells via NF-kappaB and p21 signaling pathways. *Exp Eye Res* **2019**, *180*, 174-183, doi:10.1016/j.exer.2018.12.020.
5. Pain, E.; Snowden, S.; Oddy, J.; Shinhmar, S.; Alhammad, Y.M.A.; King, J.S.; Muller-Taubenberger, A.; Williams, R.S.B. Pharmacological inhibition of ENT1 enhances the impact of specific dietary fats on energy metabolism gene expression. *Proc Natl Acad Sci U S A* **2024**, *121*, e2321874121, doi:10.1073/pnas.2321874121.
6. Ogura, J.; Yamanoi, K.; Ishida, K.; Nakamura, E.; Ito, S.; Aoyama, N.; Nakanishi, Y.; Menju, T.; Kawaguchi, K.; Hosoe, Y.; et al. A stearate-rich diet and oleate restriction directly inhibit tumor growth via the unfolded protein response. *Exp Mol Med* **2024**, doi:10.1038/s12276-024-01356-2.
7. Kim, C.R.; Kim, H.S.; Choi, S.J.; Kim, J.K.; Gim, M.C.; Kim, Y.J.; Shin, D.H. Erucamide from Radish Leaves Has an Inhibitory Effect Against Acetylcholinesterase and Prevents Memory Deficit Induced by Trimethyltin. *J Med Food* **2018**, *21*, 769-776, doi:10.1089/jmf.2017.4117.
8. Li, M.M.; Jiang, Z.E.; Song, L.Y.; Quan, Z.S.; Yu, H.L. Antidepressant and anxiolytic-like behavioral effects of erucamide, a bioactive fatty acid amide, involving the hypothalamus-pituitary-adrenal axis in mice. *Neurosci Lett* **2017**, *640*, 6-12, doi:10.1016/j.neulet.2016.12.072.
9. Maiese, K. Nicotinamide as a Foundation for Treating Neurodegenerative Disease and Metabolic Disorders. *Curr Neurovasc Res* **2021**, *18*, 134-149, doi:10.2174/1567202617999210104220334.
10. Zeisel, S.H. Nutritional importance of choline for brain development. *J Am Coll Nutr* **2004**, *23*, 621S-626S, doi:10.1080/07315724.2004.10719433.
11. Hardege, I.; Morud, J.; Yu, J.; Wilson, T.S.; Schroeder, F.C.; Schafer, W.R. Neuronally produced betaine acts via a ligand-gated ion channel to control behavioral states. *Proc Natl Acad Sci U S A* **2022**, *119*, e2201783119, doi:10.1073/pnas.2201783119.
12. Fujiwara, M.; Sato, N.; Okamoto, K. Hypoxanthine Reduces Radiation Damage in Vascular Endothelial Cells and Mouse Skin by Enhancing ATP Production via the Salvage Pathway. *Radiat Res* **2022**, *197*, 583-593, doi:10.1667/RADE-21-00223.1.
