# Supplementary material for: Chick Early Amniotic Fluid Alleviates Dextran-Sulfate-Sodium-Induced Colitis in Mice via T-Cell Receptor Pathway
Source: Antioxidants (Basel). 2025 Jan 4;14(1):51. doi: 10.3390/antiox14010051 (PMC11762673; doi:10.3390/antiox14010051)
Supplement: Supplementary file 1 [file antioxidants-14-00051-s001.zip › Supplementary Table S2.pdf]

Supplementary Table S2. Primer sequences used in quantitative RT-PCR assays.

Gene		Primer Sequences (5′-3′)	Gene Accession Number
CD3e	Forward	AAGCCTGTGACCCGAGGAACC	NM_007648.5
	Reverse	CTTTGCGGATGGGCTCATAGTCTG	
Itk	Forward	GCTCCACGGGCACCAAATTCC	NM_001281968.1
	Reverse	ACACCAAACGACCACACATCTGAC	
Cd3d	Forward	ACCGAATGTGCCAGAACTGTGTG	NM_013487.3
	Reverse	AGTAGACGCCCAAAGCCAGGAG	
CD8a	Forward	CAAATGTCCCAGGCCGCTA	NM_009857.1
	Reverse	GGCGGTGCCATTTTACACAA	
Lck	Forward	GCACGATCTAGTCCGCCATTACAC	NM_001162433.2
	Reverse	CAGCCGCTCCACCAACTTCAG	
Lat	Forward	AGACGACTATCCCAACGGCTACC	NM_010689.3
	Reverse	CTAGGCACAGGAGCAGAGGAGAC	
CD3g	Forward	CATTGCGGGACAGGATGGAGTTC	NM_009850.3
	Reverse	TCCTTGGAGATGGCTGTACTGGTC	
CD8b1	Forward	CGAAGCTGACTGTGGTTGATG	NM_009858.3
	Reverse	CAGGATGCAGACTACCAGCA	
GAPDH	Forward	GGCAAATTCAACGGCACAGTCAAG	AY618199.1
	Reverse	TCGCTCCTGGAAGATGGTGATGG	
